# Associations of LRP5 Gene With Bone Mineral Density, Bone Turnover Markers, and Fractures in the Elderly With Osteoporosis

**DOI:** 10.3389/fendo.2020.571549

**Published:** 2020-09-25

**Authors:** Qi-Fei Wang, Hong-Sen Bi, Ze-Lian Qin, Pu Wang, Fang-Fei Nie, Guang-Wu Zhang

**Affiliations:** ^1^Department of Plastic Surgery, Peking University Third Hospital, Beijing, China; ^2^Department of Orthopedics, Peking University Shougang Hospital, Beijing, China

**Keywords:** low density lipoprotein receptor-related protein 5, polymorphism, osteoporosis, bone mineral density, bone turnover, fractures

## Abstract

**Objective:** The study aimed to explore the associations of rs4988300 and rs634008 in the low-density lipoprotein receptor–related protein 5 (*LRP5*) gene with bone mineral density (BMD), bone turnover markers (BTM), and fractures in elderly patients with osteoporosis (OP).

**Methods:** Our study included 328 unrelated OP patients with or without fractures. Genomic DNA was extracted for genotyping. BTM levels were assessed by electrochemiluminescence (ECL). Dual-energy X-ray absorptiometry (DXA) was employed to measure BMD in the lumbar spine (LS) and proximal femur. Basic features between the OP and fracture groups were analyzed using the *t*-test. The Chi-square test was performed to analyze the differences in allele and genotype frequencies. The associations of single-nucleotide polymorphisms (SNPs) with BMD and BTM in the subgroups were investigated by the analysis of covariance (ANCOVA) adjusted for confounding factors.

**Results:** In both females and males, individuals with fractures exhibited higher BTM levels and lower BMD values than those with OP (*P* < 0.05). The allele and genotype frequencies of rs4988300 in the subgroups were significantly different (*P* < 0.05). In both females and males suffering from OP, participants with rs4988300 GG or rs634008 TT presented lower procollagen I N-terminal propeptide (PINP) levels (*P* < 0.05). Women with OP carrying rs4988300 GG exhibited lower BMD values at FN and TH (*P* < 0.05). In both females and males with fractures, individuals carrying rs4988300 GG genotype or rs634008 TT genotype exhibited lower PINP levels and BMD values at FN and TH than those with other genotypes (*P* < 0.05).

**Conclusions:** Rs4988300 and rs634008 polymorphisms in the *LRP5* gene are associated with bone phenotypes in the elderly with OP or fractures.

## Introduction

OP, a complex metabolic disorder distinguished by decreased bone mass, a compromised bone microarchitecture and increased fracture risk, represents a major public health issue worldwide (1). Individuals with OP are prone to suffer from osteoporotic fractures under low-energy trauma. Osteoporotic fractures, also referred to as fragility fractures, are associated with poor outcomes (2). The mortality rate in the first year after hip fracture is increased to 20% (3, 4). The development of OP is largely influenced by genetic and environmental factors (5). Genetic factors that regulate bone metabolism and affect bone mass are reported to account for ~60–80% of the variance in BMD (6), which is a major predictor of osteoporotic fractures (7).

The Wnt pathway plays a key role in bone metabolism. It influences the differentiation and function of osteoblasts and osteoclasts, and its dysregulation leads to various forms of inherited bone mass disorders (8, 9). LRP5, a component of the Wnt pathway, is a key regulator of bone mass (10, 11). Loss of function mutations in the LRP5 gene result in osteoporosis-pseudoglioma syndrome (OPPG), a condition characterized by blindness and severe osteoporosis (12, 13). Conversely, gain of function mutations of LRP5 lead to high bone mass (14, 15). In recent decades, genome-wide association studies (GWAS) focusing on the LRP5 gene have confirmed a close relationship of its genotypes with osteoporosis (16, 17). Accordingly, associations between SNPs in the *LRP5* gene and BMD or osteoporotic fractures have been extensively explored. Although the association of SNPs in the LRP5 gene with BMD was discussed previously, the findings may not be verified in other populations owing to genetic variations and multiple environmental factors. In addition, most previous studies have only involved postmenopausal women, rather than elderly individuals whose life quality would be seriously compromised when they sustain osteoporotic fractures. Furthermore, most studies have not focused on fractures when investigating the relationship between SNPs and OP. In our study, we investigated the relationships of rs4988300 and rs634008 in the LRP5 gene with BTMs, BMD, and fractures in an elderly Chinese population.

## Materials and Methods

### Subjects

All subjects were randomly enrolled from Peking University Shougang Hospital. They were unrelated individuals of Chinese Han nationality. Females aged >65 years and males aged >70 years who were diagnosed with primary osteoporosis or osteoporotic fractures under low trauma were included in the study. Participants with a history of endocrine disorders (e.g., diabetes mellitus, hyperthyroidism) or other systemic diseases such as cardiovascular, hepatic, renal, gastrointestinal tract, or rheumatologic disorders that affect bone metabolism were excluded from the study. Individuals receiving treatment with drugs (e.g., calcium, vitamin D, estrogen, bisphosphonate, corticosteroids, anticonvulsants, or calcitonin) that affect bone metabolism and BMD in the last 6 months were also excluded from the study. In addition, subjects with lifestyles including smoking, drinking, and excessive consumption of coffee and carbonated drinks that affect bone metabolism were excluded from the study. Finally, 156 patients with SOP (aged 65–90 years) and 172 participants with osteoporotic fractures (aged 65–96 years) were recruited. Each patient's weight (kg) and height squared (m^2^) were collected to calculate body mass index (BMI). Age, sex, and BMI were considered as covariates in the data analysis. Written informed consent was obtained from all participants, and the study was approved by the local ethics committee.

### BTMs Measurements

All blood samples were extracted between 7 and 9 a.m. after an overnight fast of at least 8 h. Serum was stored at −20°C until examination. The levels of BTMs including procollagen type I carboxy terminal peptide beta special sequence (β-CTX) and PINP were measured using ECL assay kits from Roche Laboratory (Mannheim, Germany) according to the manufacturer's instructions. Measurements were performed by clinical laboratory physicians.

### BMD Measurements

The measurements of the BMD (g/cm^2^) of the LS and proximal femur (including FN: femoral neck; WT: Ward's triangle; FT: femoral trochanter. TH: total hip) were performed by DXA (QDR-4500; HOLOGIC Inc., Bedford, MA, USA). The instrument was calibrated daily according to the manufacturer's instructions. Osteoporosis was considered to exist if one T-score at any site was lower than – 2.5 SD. The T-scores were the number of SDs for a normal young adult reference population. BMD values were reported as grams per cm^2^. The coefficient of variation (*CV*) was obtained from three repeated measurements from 20 participants and varied between 0.7 and 2.1%. Patients with osteoporotic fractures were given a 100 mg bucinnazine hydrochloride injection (NEPG, Chenyang, China) to relieve pain caused by BMD measurements. The determination process was assisted by clinical radiologists.

### Genotyping

Rs4988300 and rs634008 polymorphisms in the *LRP5* gene were genotyped in our study because these SNPs were reported to be the lead SNPs in an extremely large published GWAS associated with BMD. Basic information on the studied SNPs is provided in Table 1. Whole blood was extracted from the cubital veins in tubes containing disodium ethylenediaminetetraacetic acid (EDTA) and stored at 4°C until extraction. Genomic DNA was isolated from nucleated cells using the QIAamp DNA Blood Mini Kit (QIAGEN GmbH, Hilden, Germany). DNA was stored at −80°C until genotyping. SNaPshot technology was utilized for the identification of rs4988300 and rs634008 polymorphisms. Genotyping was performed by Beijing Microread Gene Technology Co., Ltd. To ensure genotyping quality, a random sample (5% of the total genotyped samples) was also repeatedly genotyped in a separate control plate with a 100% coincidence rate.

**Table 1:** Basic information of the study SNPs.

**SNP**	**Variant type**	**Alleles**	**Chromosome**	**Gene**	**Functional consequence**	**MAF**
rs4988300	SNV	G>T	11:68321363 (GRCh38) 11:68088831 (GRCh37)	LRP5	genic_upstream_transcript_variant,intron_variant	T = 0.395 367/1,980 (1000Genomes)
rs634008	SNV	C>T	11:68327273 (GRCh38) 11:68094741 (GRCh37)	LRP5	genic_upstream_transcript_variant,intron_variant	C = 0.436 302/2,185 (1000Genomes)

### Statistical Analysis

Categorical variables are reported as frequencies, and continuous variables are described as the mean ± standard deviation (SD). The deviation of the allele frequency from Hardy-Weinberg equilibrium (HWE) in all patients was tested with the χ^2^-test. A *P* > 0.05 suggested HWE. Differences in allele and genotype frequencies in the osteoporotic and fracture groups were examined by the χ^2^-test. The basic features of the osteoporotic and fracture groups were analyzed by Student's *t-*test. Variable parameters in different genotypes in the osteoporotic or fracture group were assessed with ANCOVA adjusted for confounding factors. *P* < 0.05 was considered statistically significant. Statistical analysis was performed by using SPSS software version 24.

## Results

### Basic Characteristics of the Study Population

The detailed characteristics of the study population including 328 unrelated subjects are summarized in Table 2. The study consisted of 156 subjects with OP and 172 individuals with osteoporotic fractures. The basic characteristics of the two groups, such as their age, sex, BMI, BTM levels, and BMD at each site, were also documented in our previous study (18). A lower BMD value was detected at each site in females with fractures compared with females with OP (*P* < 0.05 by *t-*test). Furthermore, individuals with fractures exhibited higher PINP levels than those with OP (*P* < 0.05 by *t-*test). Interestingly, a similar result was found between males with fractures and males with OP. Males with fractures exhibited higher PINP levels and lower BMD at each site than those with OP (*P* < 0.05 by *t-*test).

**Table 2:** Characteristics of the study population.

**Variable**	**Female**		***P***	**Male**		***P***
	**Osteoporosis**	**Fracture**		**Osteoporosis**	**Fracture**	
*N*	98	103		58	68	0.586
Age (years)	71.6 ± 12.1	75.8 ± 13.4	0.021	73.8 ± 13.2	78.5 ± 12.6	0.04
BMI (kg/m^2^)	21.303 ± 5.551	19.183 ± 4.532	0.003	22.702 ± 3.783	20.502 ± 3.541	0.031
β-CTX (ng/ml)	0.461 ± 0.098	0.467 ± 0.086	0.645	0.442 ± 0.099	0.446 ± 0.093	0.816
PINP (ng/ml)	49.231 ± 9.288	52.678 ± 9.939	0.019	51.141 ± 8.879	54.53 ± 9.117	0.037
BMD-L2-4 (g/cm^2^)	0.944 ± 0.178	0.743 ± 0.154	<0.001	1.062 ± 0.236	0.81 ± 0.18	<0.001
BMD-FN (g/cm^2^)	0.532 ± 0.108	0.47 ± 0.094	<0.001	0.658 ± 0.126	0.558 ± 0.113	0.001
BMD-WT (g/cm^2^)	0.517 ± 0.097	0.401 ± 0.075	<0.001	0.528 ± 0.121	0.416 ± 0.092	<0.001
BMD-FT (g/cm^2^)	0.643 ± 0.121	0.519 ± 0.097	<0.001	0.665 ± 0.147	0.534 ± 0.118	<0.001
BMD-TH (g/cm^2^)	0.832 ± 0.156	0.712 ± 0.134	<0.001	0.843 ± 0.182	0.729 ± 0.162	<0.001

*β-CTX, procollagen type I carboxy terminal peptide beta special sequence; PINP, procollagen I N-terminal propeptide; BMD, bone mineral density; L2-4, L2-4 vertebra; FN, femoral neck; WT, Ward's triangle; FT, femoral trochanter; TH, total hip*.

### Distribution of Allele and Genotype Frequencies

The distributions of the allele and genotype frequencies of rs4988300 and rs634008 in the control and fracture groups are described in Table 3. The genotype distributions of the two SNPs in the control group agreed closely with HWE, indicating that these participants were representatives of the local population. The allele and genotype frequency distributions of rs4988300 in the subgroups were significantly different (P-allele = 0.005, P-genotype = 0.02 tested by the χ^2^-test), suggesting a statistical correlation with fracture risk. No significant difference in the allelic and genotypic frequencies of rs634008 was found in the control and fracture groups (P-allele = 0.147, P-genotype = 0.12 using the χ^2^-test).

**Table 3:** Distributions of allele and genotype frequencies in the study sample.

**SNP**	**Group**	**Allele**		***df***	***P*-Allele**	**Genotype**			***df***	***P*-Genotype**	***P*-HWE**
		T	G			TT	TG	GG			
rs4988300	Conrol	137 (43.9%)	175 (56.1%)	1	0.005	30 (19.2%)	77 (49.4%)	49 (31.4%)	2	0.02	
	Fracture	114 (33.1%)	230 (66.9%)			19 (11.05%)	76 (44.2%)	77 (44.8%)			
	Total	251 (38.3%)	405 (61.7%)			49 (14.9%)	153 (46.7%)	126 (38.4%)			0.819
rs634008		T	C			TT	TC	CC			
	Conrol	162 (51.9%)	150 (48.1%)	1	0.147	40 (25.6%)	78 (50%)	38 (24.4%)	2	0.12	
	Fracture	198 (57.6%)	146 (42.4%)			59 (34.3%)	84 (48.9%)	29 (46.9%)			
	Total	360 (54.9%)	296 (45.1%)			99 (30.2%)	162 (49.4%)	67 (20.4%)			0.961

### Relationships of Genotyped SNPs With BTMs and BMD in Individuals With OP

The associations of SNPs with BTMs and BMD in participants with OP are shown in Tables 4, 5. A significant relationship of the genotyped SNPs with PINP levels in females and males with OP was observed after adjustment for age and BMI by ANCOVA (P-rs4988300 = 0.018 P-rs634008 = 0.032 in females with OP, P-rs4988300 = 0.037 P- rs634008 = 0.009 in males with OP). In the female population with OP, individuals with the GG genotype of rs4988300 exhibited lower BMD values of FN and TH than those with other genotypes (P-FN = 0.029, P-TH = 0.027 with adjustment for age and BMI by ANCOVA). In the male population with OP, participants carrying the rs4988300 GG genotype exhibited a lower BMD value at FN than those carrying the GT or TT genotype (*P* = 0.02 by adjusted with confounding factors).

**Table 4:** Relationships of SNPs genotyped with BTMs and BMDs in female with OP.

**Variable**	**Rs4988300**			***P***	**Rs634008**			***P***
	**GG**	**GT**	**TT**		**TT**	**TC**	**CC**	
β-CTX (ng/ml)	0.475 ± 0.104	0.467 ± 0.095	0.423 ± 0.094	0.164	0.476 ± 0.124	0.465 ± 0.135	0.437 ± 0.105	0.529
PINP (ng/ml)	46.542 ± 6.634	50.057 ± 6.21	51.532 ± 6.824	0.018	46.652 ± 6.584	49.438 ± 6.372	51.495 ± 6.214	0.032
BMD-L2-4 (g/cm^2^)	0.921 ± 0.178	0.952 ± 0.167	0.961 ± 0.183	0.66	0.882 ± 0.192	0.954 ± 0.187	0.988 ± 0.175	0.123
BMD-FN (g/cm^2^)	0.532 ± 0.113	0.598 ± 0.105	0.597 ± 0.124	0.029	0.554 ± 0.124	0.578 ± 0.132	0.599 ± 0.118	0.464
BMD-WT (g/cm^2^)	0.504 ± 0.098	0.522 ± 0.115	0.526 ± 0.108	0.718	0.504 ± 0.114	0.523 ± 0.105	0.518 ± 0.124	0.788
BMD-FT (g/cm^2^)	0.638 ± 0.124	0.646 ± 0.134	0.653 ± 0.135	0.922	0.635 ± 0.127	0.642 ± 0.119	0.653 ± 0.134	0.878
BMD-TH (g/cm^2^)	0.784 ± 0.132	0.847 ± 0.116	0.872 ± 0.122	0.027	0.801 ± 0.135	0.838 ± 0.142	0.852 ± 0.132	0.398

*β-CTX, procollagen type I carboxy terminal peptide beta special sequence; PINP, procollagen I N-terminal propeptide; BMD, bone mineral density; L2-4, L2-4 vertebra; FN, femoral neck; WT, Ward's triangle; FT, femoral trochanter; TH, total hip. All P-values were adjusted for age and BMI by ANCOVA*.

**Table 5:** Relationships of SNPs genotyped with BTMs and BMDs in male with OP.

**Variable**	**Rs4988300**			***P***	**Rs634008**			***P***
	**GG**	**GT**	**TT**		**TT**	**TC**	**CC**	
β-CTX (ng/ml)	0.466 ± 0.084	0.447 ± 0.078	0.432 ± 0.074	0.523	0.46 ± 0.084	0.439 ± 0.075	0.446 ± 0.072	0.692
PINP (ng/ml)	46.835 ± 6.872	51.124 ± 6.847	53.8 ± 6.895	0.037	47.027 ± 6.749	51.427 ± 6.814	54.956 ± 6.452	0.009
BMD-L2-4 (g/cm^2^)	0.991 ± 0.174	1.063 ± 0.172	1.104 ± 0.178	0.244	1.022 ± 0.182	1.104 ± 0.174	1.018 ± 0.192	0.217
BMD-FN (g/cm^2^)	0.522 ± 0.125	0.635 ± 0.127	0.659 ± 0.134	0.02	0.569 ± 0.142	0.632 ± 0.148	0.654 ± 0.136	0.247
BMD-WT (g/cm^2^)	0.51 ± 0.114	0.526 ± 0.108	0.542 ± 0.117	0.755	0.518 ± 0.104	0.53 ± 0.11	0.535 ± 0.106	0.909
BMD-FT (g/cm^2^)	0.639 ± 0.134	0.663 ± 0.128	0.674 ± 0.142	0.79	0.648 ± 0.138	0.669 ± 0.127	0.675 ± 0.141	0.841
BMD-TH (g/cm^2^)	0.776 ± 0.145	0.848 ± 0.164	0.876 ± 0.162	0.265	0.806 ± 0.162	0.847 ± 0.168	0.874 ± 0.171	0.541

*β-CTX, procollagen type I carboxy terminal peptide beta special sequence; PINP, procollagen I N-terminal propeptide; BMD, bone mineral density; L2-4, L2-4 vertebra; FN, femoral neck; WT, Ward's triangle; FT, femoral trochanter; TH, total hip. All P-values were adjusted for age and BMI by ANCOVA*.

### Associations of Genotyped SNPs With BTMs and BMD in Participants With Osteoporotic Fractures

The associations of rs4988300 and rs634008 polymorphisms with BTMs and BMD values among the study participants with osteoporotic fractures are presented in Tables 6, 7. With regard to rs4988300, PINP levels, and BMD values at FN and TH in females suffering from osteoporotic fractures carrying the GG genotype were lower than in those carrying GT or TT after adjustment for age and BMI by ANCOVA (P-PINP = 0.014, P- FN < 0.001, and P-TH < 0.001). For rs634008, female participants carrying the TT genotype exhibited a lower PINP concentration and BMD values of FN and TH following adjustment for age and BMI by ANCOVA (P-PIN*P* < 0.001, P- FN < 0.001, and P-TH = 0.002). In addition, we analyzed the association of study SNPs with phenotypes in the males with osteoporotic fractures. Subjects carrying the GG genotype of rs4988300 exhibited lower PINP levels and BMD values at FN and TH after adjustment for age and BMI by ANCOVA (P-PINP = 0.004, P- FN = 0.02, and P-TH = 0.025). Concerning rs634008, lower PINP levels, and BMD values at FN and TH were detected in males suffering from osteoporotic fractures carrying the TT genotype (P-PIN*P* < 0.001, P- FN = 0.006, and P-TH = 0.029 after adjustment for age and BMI).

**Table 6:** Relationships of SNPs genotyped with BTMs and BMDs in female with osteoporotic fracture.

**Variable**	**Rs4988300**			***P***	**Rs634008**			***P***
	**GG**	**GT**	**TT**		**TT**	**TC**	**CC**	
β-CTX (ng/ml)	0.472 ± 0.106	0.442 ± 0.095	0.444 ± 0.092	0.628	0.463 ± 0.088	0.44058 ± 0.082	0.428 ± 0.093	0.312
PINP (ng/ml)	45.365 ± 7.35	51.239 ± 7.19	52.451 ± 6.87	0.014	47.275 ± 7.47	52.263 ± 7.823	55.541 ± 7.828	<0.001
BMD-L2-4 (g/cm^2^)	0.995 ± 0.184	1.102 ± 0.198	1.037 ± 0.183	0.126	1.024 ± 0.173	1.073 ± 0.185	1.104 ± 0.184	0.264
BMD-FN (g/cm^2^)	0.534 ± 0.083	0.628 ± 0.072	0.635 ± 0.062	<0.001	0.552 ± 0.082	0.63546 ± 0.075	0.715 ± 0.086	<0.001
BMD-WT (g/cm^2^)	0.487 ± 0.068	0.538 ± 0.087	0.528 ± 0.074	0.166	0.513 ± 0.074	0.53 ± 0.078	0.551 ± 0.072	0.22
BMD-FT (g/cm^2^)	0.628 ± 0.058	0.664 ± 0.078	0.675 ± 0.062	0.136	0.652 ± 0.074	0.667 ± 0.086	0.685 ± 0.082	0.37
BMD-TH (g/cm^2^)	0.735 ± 0.087	0.843 ± 0.093	0.869 ± 0.108	<0.001	0.797 ± 0.109	0.856 ± 0.094	0.896 ± 0.102	0.002

*β-CTX, procollagen type I carboxy terminal peptide beta special sequence; PINP, procollagen I N-terminal propeptide; BMD, bone mineral density; L2-4, L2-4 vertebra; FN, femoral neck; WT, Ward's triangle; FT, femoral trochanter; TH, total hip. All P-values were adjusted for age and BMI by ANCOVA*.

**Table 7:** Relationships of SNPs genotyped with BTMs and BMDs in male with osteoporotic fracture.

**Variable**	**Rs4988300**			***P***	**Rs634008**			***P***
	**GG**	**GT**	**TT**		**TT**	**TC**	**CC**	
β-CTX (ng/ml)	0.46 ± 0.072	0.451 ± 0.067	0.438 ± 0.064	0.613	0.452 ± 0.071	0.449 ± 0.063	0.424 ± 0.058	0.461
PINP (ng/ml)	46.152 ± 7.239	54.621 ± 7.873	56.542 ± 7.452	0.004	49.127 ± 7.658	56.874 ± 7.318	59.073 ± 7.641	<0.001
BMD-L2-4 (g/cm^2^)	0.743 ± 0.142	0.802 ± 0.153	0.834 ± 0.157	0.311	0.778 ± 0.165	0.814 ± 0.162	0.867 ± 0.163	0.325
BMD-FN (g/cm^2^)	0.544 ± 0.122	0.668 ± 0.124	0.684 ± 0.125	0.02	0.588 ± 0.139	0.692 ± 0.135	0.724 ± 0.132	0.006
BMD-WT (g/cm^2^)	0.403 ± 0.075	0.412 ± 0.073	0.423 ± 0.074	0.733	0.407 ± 0.069	0.419 ± 0.072	0.426 ± 0.073	0.719
BMD-FT (g/cm^2^)	0.502 ± 0.093	0.523 ± 0.096	0.552 ± 0.104	0.333	0.513 ± 0.096	0.532 ± 1.021	0.586 ± 0.091	0.962
BMD-TH (g/cm^2^)	0.622 ± 0.132	0.711 ± 0.142	0.772 ± 0.147	0.025	0.672 ± 0.134	0.745 ± 0.142	0.804 ± 0.151	0.029

*β-CTX, procollagen type I carboxy terminal peptide beta special sequence; PINP, procollagen I N-terminal propeptide; BMD, bone mineral density; L2-4, L2-4 vertebra; FN, femoral neck; WT, Ward's triangle; FT, femoral trochanter; TH, total hip. All P-values were adjusted for age and BMI by ANCOVA*.

## Discussion

There is increasing evidence regarding the key role of the *LRP5* gene in regulating bone metabolism (10, 19). Here, we assessed the relationships of rs4988300 and rs634008 polymorphisms in the LRP5 gene with BTM, BMD values and fractures in a study population with SOP or osteoporotic fractures. Interestingly, in both females and males with fractures, individuals carrying the rs4988300 GG genotype or the rs634008 TT genotype exhibited lower BTM levels and BMD values than those with other genotypes. In addition, in both females and males suffering from OP, participants with rs4988300 GG or rs634008 TT presented lower PINP levels. Women with OP carrying rs4988300 GG exhibited lower BMD values at FN and TH. Furthermore, a significant difference in the distribution of the allele and genotype frequencies of rs4988300 between the fracture and osteoporotic groups was detected.

Primary OP, including postmenopausal OP (PMOP; type I) and SOP (type II) (20), represents a major public health issue in both developed and developing countries. In contrast to PMOP, mainly owing to estrogen withdrawal, SOP generally refers to OP in females aged >65 years or males aged >70 years and is largely affected by aging (18). Hence, the pathogenesis of OP in aging women and elderly men is different. Bone mass loss in elderly women is largely affected by aging and gonadal hormone levels, while the SOP in men is mainly determined by aging. Accordingly, it is necessary for the control and fracture groups to be further stratified by sex.

The pathogenesis of OP is largely determined by genetic factors. Numerous studies have demonstrated that genetic variances in the estrogen receptor (*ER*) gene (21, 22), vitamin D receptor (*VDR*) gene (23, 24), and genes of the *RANKL-RANK-OPG* system (25) are implicated in regulating bone metabolism and influencing bone mass. Additionally, LRP5, a co-receptor of the Wnt pathway, was previously reported as a potential factor in the development of OP (26). Loss of function mutations in the *LRP5* gene result in OPPG. In contrast, gain of function mutations of *LRP5* lead to high bone mass. Importantly, many SNPs in the LRP5 gene are reported to be associated with BMD and osteoporotic fractures (15, 17, 27). Although rs4988300 and rs634008 are related to decreased bone mass and osteoporotic fractures, these findings may not be found in other ethnicities and regions, which is mainly explained by the heterogeneity of study populations. Furthermore, their relationships with BTMs have not been investigated. Accordingly, further studies are needed that focus on the relationships of SNPs in the LRP5 gene with BMD, BTMs and fractures in other races and regions, especially in the population with SOP.

In this study, we explored whether the genotypes of rs4988300 and rs634008 in the control group conformed to HWE. Interestingly, *P* > 0.05 was detected for both genotypes, suggesting that all subjects were representatives of the local population. Furthermore, significant differences in the distributions of alleles and genotypes of rs4988300 in subgroups were found (P-allele = 0.005; P-genotype = 0.02). The GG genotype frequency in the fracture group was higher than that in the control group. In addition, no remarkable difference in the allele frequencies of rs4988300 was found between our study and 1000 Genomes data (G = 0.395/1980) (*P* = 0.531). Interestingly, we detected a significant difference in rs4988300 allele frequencies between our study and Horváth's study (G = 0.463/823) (*P* < 0.001) (28). The significant difference may be explained by racial diversity. However, with regarding to rs634008, the allele frequencies observed in the present study were similar to those in the 1000 Genomes database (G = 0.463/2185) (*P* = 0.469) and Horváth's report (G = 0.443/788) (*P* = 0.724).

Bone remodeling comprises two process, bone resorption and bone formation (29). Serum β-CTX and PINP are recommended as markers of bone resorption and bone information by the International Osteoporosis Foundation (IOF) and International Federation of Clinical Chemistry (30). Although BTMs are not used as a tool for the diagnosis of osteoporosis and improved prediction of bone loss or fractures, they are useful for the assessment of the response to anabolic and antiresorptive therapies, compliance with therapy and the bone safety of new medications (30, 31). In our study, we investigated the rs4988300 and rs634008 polymorphisms in the LRP5 gene together with serum β-CTX and PINP levels in all participants. Interestingly, we found that in both the female and male population, the PINP levels of patients with the GG genotype of rs4988300 and TT genotype of rs634008 were lower than in patients with the other genotypes. However, no significant difference in β-CTX levels was observed among individuals with different genotypes of rs4988300 or rs634008. The above findings indicated that variants in the LRP5 gene regulate bone metabolism largely by affecting bone information. Additionally, studies focusing on the association of rs4988300 and rs634008 polymorphisms with BTM concentrations have not been reported previously.

According to the guidelines for OP diagnosis, BMD still represents the “gold standard” for the diagnosis of OP and fracture risk prediction (32). Accordingly, BMD is considered as a primary phenotype in studies focusing on the relationships of gene polymorphism with OP (33, 34). A recent report by Horváth et al. (28) concluded that rs4988300 and rs634008 were statistically associated with the BMD of the hip in a cohort of Caucasian postmenopausal women, but the association of rs634008 with the BMD value of the hip lost significance after Bonferroni correction. Xiong et al. (35) reported that rs4988300 was related to spine BMD and that rs634008 showed a relationship with BMD at the spine, hip and ultradistal radius. Our results were similar to Horváth's report. Individuals with the rs4988300 GG genotype or rs634008 TT genotype exhibited lower BTM levels and BMD values than those carrying other genotypes in both females and males with fractures. In addition, women with OP carrying rs4988300 GG presented lower BMD values at FN and TH. In males with OP, individuals carrying the rs4988300 GG genotype presented a lower BMD value at FN. Different from Xiong' s study results, no significant association of the studied SNPs with BMD at LS was observed, which could be attributed to genetic heterogeneity in different study populations and gene-gene interactions.

Our study has some potential limitations. First, the study population was of intermediate size. Hence, the statistical power to observe genotype–phenotype associations may be compromised. Second, elderly individuals without OP were not enrolled because there were so few participants with normal bone mass or osteopenia, especially above 75 years of age, who matched the fracture or osteoporotic group in terms of age and BMI. Basic data on the number of pregnancies, breastfeeding, dietary calcium, and vitamin D intake and life-style related variables, including smoking, alcohol intake, and physical activities, were not included in the analysis. In our future work, we will take into consideration the above risk factors for OP. Additionally, we did not determine the relationship of the haplotypes formed by SNPs with clinical phenotypes in the present study.

Our results conclusively indicate that SNPs in the LRP5 gene are involved in regulating bone metabolism and affecting bone mass. The distribution of the allele and genotype frequencies of rs4988300 was significantly different between subgroups, suggesting that the G allele is a factor in fracture risk. In addition, the GG genotype of rs4988300 and the TT genotype of rs634008 were associated with decreased PINP levels and BMD values at FN and TH.

## Data Availability Statement

The data used to support the findings of this study are available from the corresponding author upon request.

## Ethics Statement

The studies involving human participants were reviewed and approved by ethical committee of Peking University Shougang Hospital. The patients/participants provided their written informed consent to participate in this study.

## Author Contributions

Q-FW collected the data, performed statistical analysis, and drafted the research paper. H-SB revised the manuscript. G-WZ conceived and designed the experiments. PW and F-FN acquired and analyzed the data. Z-LQ and G-WZ were responsible for its financial supports and the corresponding works. All authors contributed to the article and approved the submitted version.

## Conflict of Interest

The authors declare that the research was conducted in the absence of any commercial or financial relationships that could be construed as a potential conflict of interest.
